# Supplementary material for: Morbidity of Returning Travelers Seen in Community Urgent Care Centers throughout Israel
Source: Trop Med Infect Dis. 2023 Jun 13;8(6):319. doi: 10.3390/tropicalmed8060319 (PMC10301267; doi:10.3390/tropicalmed8060319)
Supplement: Supplementary file 1 [file tropicalmed-08-00319-s001.zip › tropicalmed-2365671-supplementary.pdf]

Tropical Medicine and Infectious Disease

Communication

Morbidity of Returning Travelers Seen in Community Urgent Care Centers throughout Israel †

Eyal Itzkowitz [1,‡], Evan A. Alpert [2,3,‡], Abdulhadi Z. Farojeh [4], Deena R. Zimmerman [4,5], Eli Schwartz [6,7,*] and Tamar Lachish [3,8]

Supplement S1. Detecting travelers - Electronic questionnaire for TEREM clinics

Clerk

- Have you been abroad in the last month? YES/ NO

Nurse/ Physician

- Demographics - Age (drop down list), Sex (drop down list), Country of birth (drop down list)
- Medical history (dialog box)
- Departure date (drop down list), return date (drop down list)
- Continents visited (drop down list), countries visited (drop down list)
- Nature of travel (drop down list)
- Vaccination history before travel YES/ NO
- Medical consultation before travel YES/ NO
- Does the patient think that his/ her illness is travel related? YES/ NO/ UNSURE
- Physician opinion on the same question YES/ NO/ UNSURE
- Diagnosis (drop down list)
- Diagnosis category (medical/ trauma)
- Outcome (discharge, referral to ER, ambulance evacuation, death)
- Infectious diseases consultation YES/ NO
